# Sex hormone-specific regulation of ferroptosis in vascular cells in atherosclerosis: molecular mechanisms and targeted strategies

**DOI:** 10.3389/fphys.2025.1680625

**Published:** 2025-10-01

**Authors:** Keying Yu, Jitong Li, Tenghui Tian, Rui Shi, Yue Deng, Liping Chang

**Affiliations:** ^1^ Affiliated Hospital of Changchun University of Chinese Medicine, Changchun, China; ^2^ Changchun University of Chinese Medicine, Changchun, China; ^3^ Jilin Provincial Traditional Chinese Medicine Cardiovascular Disease Research Center, Changchun, China

**Keywords:** atherosclerosis, ferroptosis, sex hormones, estrogen, androgen, sex differences, precision medicine, vascular cells

## Abstract

Atherosclerosis (AS), the leading cause of cardiovascular morbidity and mortality worldwide, exhibits significant sex differences in its incidence and pathological progression, yet the underlying molecular mechanisms remain fully elucidated. Ferroptosis, a form of regulated cell death driven by iron-dependent lipid peroxidation, has recently been identified as a key pathological event contributing to the progression of AS. The basis of physiological sex dimorphism is composed of both circulating sex hormone levels and cell-intrinsic sex differences, which may play a critical role in determining the sex-specific characteristics of AS by modulating the ferroptosis signaling network. This review aims to systematically elaborate and substantiate the “sex hormone-ferroptosis regulatory axis” as a pivotal theoretical framework in the context of AS-related sex differences. We integrate existing evidence suggesting that estrogen can synergistically inhibit ferroptosis in vascular cells, particularly endothelial cells and macrophages, through multiple pathways. These include: (1) activating the central antioxidant system driven by Nuclear Factor Erythroid 2-Related Factor 2 (Nrf2); (2) regulating mitochondrial homeostasis and function; and (3) directly modulating key iron metabolism proteins, such as upregulating the iron efflux protein Ferroportin-1 (FPN1). These mechanisms collectively contribute to the cardiovascular protective effects observed in premenopausal women. Conversely, available evidence suggests that androgens may promote ferroptosis in vascular cells by enhancing oxidative stress, potentially increasing cellular iron uptake (e.g., through potential upregulation of Transferrin Receptor 1, TFR1), and modulating lipid metabolism to increase the availability of peroxidizable substrates. This could be a significant contributor to the earlier onset and higher incidence of AS in men. Based on this framework, this review further explores potential sex-specific therapeutic strategies targeting this regulatory axis. This review provides a novel molecular perspective for understanding the sex differences in AS and provides a theoretical basis for the development of a new paradigm in sex-stratified precision cardiovascular medicine.

## 1 Introduction

Atherosclerosis (AS) and its complications are the leading cause of death worldwide, and its epidemiological characteristics reveal significant sex dimorphism. Clinical data indicate that even after adjusting for traditional risk factors, the age of onset of AS in men is significantly earlier than in women (Swahn et al., 2024). However, after menopause, as estrogen levels sharply decline, the protective effect on the cardiovascular system in women rapidly diminishes, leading to a steep increase in the incidence and mortality of AS, which gradually equals or even exceeds that of men of the same age. This strong sex- and age-dependent pattern clearly indicates that sex-related biological factors play a key regulatory role in the pathophysiology of AS. These factors include not only differences in circulating sex hormone levels but also cell-intrinsic biological differences determined by sex chromosomes.

Recent discoveries in cell biology have revealed a novel form of regulated cell death—ferroptosis. Ferroptosis is driven by iron-dependent, membrane lipid peroxidation and is morphologically, biochemically, and genetically distinct from apoptosis, necrosis, and autophagic cell death (Dixon et al., 2012). In the pathological environment of AS plaques, local hypoxia, inflammatory cell infiltration, high levels of oxidative stress, and abnormal lipid accumulation create a conducive environment for ferroptosis to occur. A growing body of direct evidence indicates that ferroptosis of key vascular cells, including vascular endothelial cells, vascular smooth muscle cells (VSMCs), and macrophages, is critically implicated in the pathological progression of AS, from early endothelial dysfunction and foam cell formation to late-stage necrotic core expansion and fibrous cap thinning and rupture (Xu et al., 2024; Jinso et al., 2025). For example, in a deep vein thrombosis model, ferroptosis of endothelial progenitor cells was identified as a key event affecting vascular recanalization, a process involving key molecules like lipoxygenase (ALOX15), highlighting the important role of ferroptosis in vascular pathophysiology (Li et al., 2025). Additionally, adaptive coatings for vascular stents that control atherosclerosis by modulating coagulation-inflammation to support re-endothelialization also indirectly underscore the importance of inflammation and endothelial cell function in AS (Wang Y. et al., 2025).

Connecting the significant sex differences in AS with this recently identified cell death modality of ferroptosis leads to a critical scientific question: Do sex hormones, as major biological determinants of sexual dimorphism, influence the sex-specific clinical progression of AS by directly or indirectly regulating the molecular machinery of ferroptosis? Although the macroscopic effects of sex hormones on the cardiovascular system (Swahn et al., 2024) and the independent role of ferroptosis in AS (Xu et al., 2024; Jinso et al., 2025) have been extensively studied, there is a scarcity of research that systematically elucidates the intrinsic molecular links between the two. For example, evidence has shown that low testosterone levels in men are significantly associated with endothelial dysfunction and increased arterial stiffness (Sakamuri et al., 2024), while other studies have observed a close relationship between sex hormone levels and markers of vascular inflammation (Souza et al., 2023). These findings provide evidence that sex hormones regulate the progression of AS by influencing vascular function and inflammation.

This review aims to systematically organize and critique existing evidence to construct a theoretical framework for the “sex hormone-ferroptosis regulatory axis” and to clarify its potential role in mediating the sexual dimorphism of AS. We will focus on how estrogen and androgens differentially regulate the ferroptosis pathway in key vascular cells (endothelial cells, macrophages, and smooth muscle cells). This review aims to provide an integrated molecular mechanistic perspective for understanding sex differences in AS and to offer a theoretical basis for exploring potential sex-specific therapeutic targets, thereby advancing the prevention and treatment of cardiovascular diseases toward a more precise approach. It is important to clarify that while cell-intrinsic genetic differences are also a crucial determinant of sexual dimorphism, this review will primarily focus on a detailed examination of sex hormones as a key endocrine regulatory layer.

## 2 Core molecular regulatory network of ferroptosis

**FIGURE 1:**
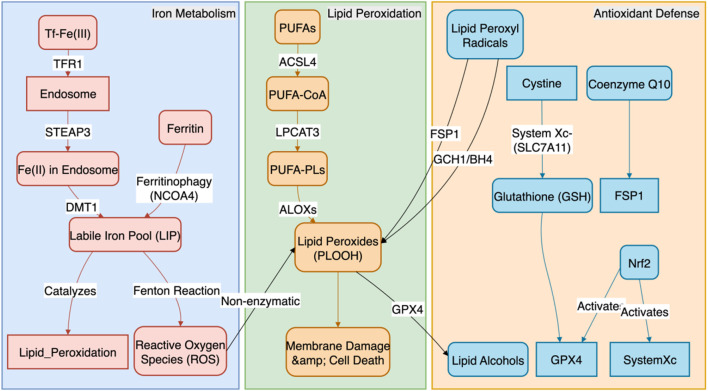
The Core Molecular Regulatory Network of Ferroptosis. This figure systematically illustrates the molecular regulatory mechanisms of ferroptosis, the core of which is a dynamic balance between pro-ferroptotic and anti-ferroptotic systems. The pro-ferroptotic pathway primarily involves two processes: (1) iron metabolism imbalance, where cells increase iron uptake via TFR1 and release stored iron through NCOA4-mediated ferritinophagy, leading to the expansion of the labile iron pool (LIP) and subsequent generation of reactive oxygen species (ROS) via the Fenton reaction; and (2) lipid peroxidation, where polyunsaturated fatty acid phospholipids (PUFA-PLs), synthesized by ACSL4 and LPCAT3, are oxidized by ALOXs or ROS into phospholipid hydroperoxides (PLOOH), ultimately causing cell membrane rupture. In contrast, the anti-ferroptotic pathway comprises three main defense systems: (1) the GPX4-centered pathway, which utilizes glutathione (GSH) to reduce PLOOH to non-toxic substances; (2) the GPX4-independent FSP1/CoQ10/BH4 pathway, which traps lipid radicals; and (3) the upstream master regulator Nrf2, which activates multiple anti-ferroptotic genes to build a systemic defense.

The occurrence of ferroptosis is the result of a dynamic balance between pro-ferroptotic forces (iron homeostasis imbalance, lipid peroxidation) and anti-ferroptotic defenses (antioxidant systems). The core biochemical event is the iron-dependent, cumulative peroxidation of polyunsaturated fatty acid (PUFA) chains in membrane phospholipids, ultimately leading to the physical rupture of the cell membrane (Figure 1). Related research has revealed the therapeutic prospects of targeting cell death in sarcopenia (Peng J. et al., 2025).

### 2.1 Iron homeostasis and the supply of catalytic iron ions

The initiation of ferroptosis is highly sensitive to the expansion of the intracellular labile iron pool (LIP). The sources and metabolism of cellular iron are tightly regulated:

1. Iron Uptake: Cells primarily internalize transferrin-bound iron (Tf-Fe^3+^) from the circulation via the transferrin receptor 1 (TFR1) on the plasma membrane. After endosomal acidification, Fe^3+^ is reduced to Fe^2+^ by the STEAP3 metalloreductase and subsequently released into the cytoplasm via the divalent metal transporter 1 (DMT1). DMT1 can also directly transport non-transferrin-bound iron. Mitochondria play a key role in processes such as iron-sulfur cluster synthesis, and their dysfunction can also affect the LIP (Ward and Cloonan, 2019). For example, in vascular lesions of patients with thromboangiitis obliterans, an inflammatory vascular disease with pathological features similar to AS, a significant upregulation of iron overload and TFR1 expression has been observed, directly linking iron uptake imbalance to vascular injury (Deng et al., 2025).
2. Iron Storage and Release: Under physiological conditions, excess iron is safely oxidized and stored in cytosolic ferritin complexes (composed of heavy-chain FTH1 and light-chain FTL). However, under ferroptosis-inducing conditions, the nuclear receptor coactivator 4 (NCOA4), a selective autophagy receptor, recognizes and binds to FTH1, mediating the degradation of ferritin in lysosomes (a process known as “ferritinophagy”). This releases large amounts of stored iron into the LIP, providing ample Fe^2+^ substrate for the Fenton reaction (Mancias et al., 2014).
3. Iron Export: The cellular iron export protein, ferroportin-1 (FPN1), is the only known protein capable of pumping intracellular Fe^2+^ out of the cell. The expression and activity of FPN1 are key determinants of cellular iron load, and its enhanced function can effectively reduce LIP levels, exerting a significant anti-ferroptotic effect (Donovan et al., 2000). The stability of FPN1 is negatively regulated by hepcidin. Notably, ferroptosis-mediated endothelial dysfunction has been confirmed as a key event in promoting thrombosis, which provides a direct link for understanding how iron homeostasis imbalance (including impaired iron export) drives vascular pathology (Karbakhsh Ravari et al., 2025).

### 2.2 Substrates and execution of lipid peroxidation

Accumulated phospholipid hydroperoxides (PLOOH) are the direct effector molecules of ferroptosis.

1. Substrate Synthesis: The specificity of this process lies in its primary attack on membrane phospholipids containing polyunsaturated fatty acids (PUFA-PLs), particularly those rich in arachidonic acid (AA) and adrenic acid (AdA) in phosphatidylethanolamine (PE). Long-chain acyl-CoA synthetase 4 (ACSL4) preferentially activates AA and AdA, while lysophosphatidylcholine acyltransferase 3 (LPCAT3) esterifies the activated PUFA-CoA into membrane phospholipids. These two enzymatic reactions together provide the necessary lipid substrates for ferroptosis (Doll et al., 2017).
2. Peroxidation Execution: In the presence of Fe^2+^ as a catalyst, these PUFA-PLs undergo stereospecific peroxidation catalyzed by members of the lipoxygenase (ALOXs) family (e.g., ALOX15, ALOX12, ALOX5), forming PLOOH. The generation of these PLOOH further triggers an uncontrolled free-radical chain reaction that rapidly spreads across the membrane, ultimately leading to the destruction of the cell membrane’s structural integrity, increased permeability, and eventual rupture (Probst et al., 2017). For instance, in chronic inflammatory diseases such as atherosclerosis, inhibiting ferroptosis by targeting key regulators like ACSL4 has been shown to be an effective therapeutic strategy for modulating lipid peroxidation and inflammatory responses (Zhang et al., 2025).

### 2.3 Multi-level antioxidant defense systems

To counter the lethal threat of lipid peroxidation, cells have evolved at least three major and synergistic antioxidant defense pathways.

1. The GPX4/GSH/System Xc^−^ Axis: This is the most central anti-ferroptosis pathway. Glutathione peroxidase 4 (GPX4) is the only selenoprotein that can use glutathione (GSH) as a cofactor to directly reduce PLOOH on the membrane to harmless phospholipid alcohols (PLOH). Inhibition of GPX4 activity or expression is the most direct way to induce ferroptosis. The function of GPX4 is entirely dependent on an adequate supply of intracellular GSH. The synthesis of GSH is closely linked to the function of the cystine/glutamate antiporter on the plasma membrane (System Xc^−^), which consists of the functional subunit SLC7A11 and the regulatory subunit SLC3A2. This transporter is responsible for supplying the key substrate, cystine, for GSH synthesis (Chen et al., 2025). In atherosclerosis models, stabilizing the expression and activity of GPX4 through signaling pathways such as Nrf2 has been proven to be a key mechanism for inhibiting macrophage ferroptosis and thus mitigating AS lesions (Wu et al., 2025; Zhu et al., 2025).
2. The FSP1/CoQ10/NAD(P)H Parallel Pathway: Ferroptosis suppressor protein 1 (FSP1), also known as AIFM2, acts as a CoQ10 oxidoreductase on the plasma membrane, functioning independently of GPX4. It utilizes NAD(P)H to reduce ubiquinone (CoQ10) to ubiquinol (CoQ10H_2_), which is a potent radical-trapping antioxidant that can directly scavenge lipid peroxyl radicals, thereby halting the propagation of the peroxidation chain reaction (Tan et al., 2025).
3. The GCH1/BH4/DHFR Supplementary Pathway: GTP cyclohydrolase 1 (GCH1) is the rate-limiting enzyme in the synthesis of tetrahydrobiopterin (BH4). Both BH4 and its regenerated dihydro form (BH2), produced via dihydrofolate reductase (DHFR) after oxidation, are potent endogenous antioxidants. They not only scavenge free radicals directly but also promote the *de novo* synthesis of CoQ10 and protect PUFAs from oxidation, thus inhibiting ferroptosis from multiple angles (Min et al., 2025).
4. Nrf2: The Master Transcriptional Regulator: As the upstream master regulator of these antioxidant defense systems, Nuclear Factor Erythroid 2-Related Factor 2 (Nrf2) plays a central role in sensing and responding to oxidative stress and electrophilic signals. Under stress conditions, Nrf2 is liberated from its inhibitor Keap1, translocates to the nucleus, and initiates the transcription of hundreds of genes containing Antioxidant Response Elements (AREs). In the context of anti-ferroptosis, Nrf2’s target genes include not only the iron storage proteins FTH1 and FTL and the System Xc^−^ subunit SLC7A11, but also enzymes involved in GSH synthesis and regeneration (GCLC, GCLM) and heme oxygenase-1 (HMOX1), thereby systematically enhancing the cell’s capacity to resist ferroptosis (Hu et al., 2026; Cui et al., 2025). In the field of atherosclerosis, activating the Nrf2 pathway has been confirmed as a key therapeutic strategy to inhibit macrophage ferroptosis and thereby reduce AS lesions, with its downstream effectors including core anti-ferroptotic proteins like GPX4 and HMOX1 (Wu et al., 2025; Zhu et al., 2025). These studies underscore the importance of the Nrf2 pathway in vascular protection and offer new directions for targeted therapies.

## 3 Core role of the “sex hormone-ferroptosis axis” in sex differences in atherosclerosis

Sex hormones, primarily estrogen (represented by 17β-estradiol, E2) and androgens (represented by testosterone), precise fine, multidimensional, and often opposing sex-specific regulation on the molecular network of ferroptosis at both transcriptional and non-transcriptional levels through their respective nuclear receptors (ERs and AR) and membrane receptor-mediated non-genomic effects (Table 1).

**TABLE 1:** Differential regulation of vascular cell ferroptosis by sex hormones and its functional impact in AS.

Regulatory level	Estrogen (primarily E2)	Androgen (primarily testosterone)	Key references
Core effect	Inhibition of ferroptosis (protective)	Promotion of ferroptosis (deleterious, speculated)	
Antioxidant system	Activates Nrf2 pathway: upregulates GPX4, FTH1, SLC7A11, etc., to systemically enhance antioxidant capacity	Exacerbates oxidative stress: may upregulate NOX activity, increasing ROS production and consuming GSH.	Wu et al. (2025), Zhu et al. (2025)
Iron metabolism	Promotes iron efflux: upregulates macrophage FPN1 expression, reducing intracellular iron Inhibits iron release (Speculated): may inhibit NCOA4-mediated ferritinophagy	Promotes iron uptake (speculated): may upregulate TFR1 expression, increasing intracellular iron	Deng et al. (2025), Qiu et al. (2025)
Mitochondrial function	Maintains mitochondrial homeostasis: promotes mitochondrial biogenesis and function via the PGC-1α axis and clears damaged mitochondria through mitophagy	Impairs mitochondrial function (speculated): associated with mitochondrial dysfunction and increased oxidative stress	Sasaki et al. (2021), Rovira-Llopis et al. (2017)
Lipid metabolism	Regulates lipid profile: lowers LDL-C, raises HDL-C, potentially reducing PUFA-PL substrates	Alters lipid profile (speculated): may increase pro-atherogenic lipids, providing more substrates for ferroptosis	Tai et al. (2025), Cai et al. (2023)
Net impact on AS	Delays AS progression: protects endothelium, inhibits macrophage foam cell formation and necrosis, stabilizes plaques	Accelerates AS progression: promotes endothelial dysfunction, exacerbates inflammation and necrotic core formation, may lead to plaque instability	Swahn et al. (2024), Peng X et al. (2025)

### 3.1 Multi-pathway synergistic inhibition of vascular cell ferroptosis by estrogen

A large body of evidence indicates that estrogen is a key protective factor for the cardiovascular system in premenopausal women. Its protective mechanisms extend beyond traditional lipid regulation and vasodilation, acting at the level of cell death by inhibiting ferroptosis in vascular cells through multiple synergistic pathways.

1. Systemic Activation of the Nrf2 Master Antioxidant Pathway: This is a core mechanism of estrogen’s anti-ferroptotic action. E2, through the classical estrogen receptor ERα, can activate upstream PI3K/Akt or ERK/MAPK signaling pathways, leading to the phosphorylation of Nrf2 or its inhibitory protein Keap1. This results in Nrf2 nuclear translocation and the synergistic upregulation of multiple core anti-ferroptosis genes, including GPX4, SLC7A11, and FTH1, thereby comprehensively enhancing the cell’s antioxidant defense capabilities (Chen et al., 2025; Cui et al., 2025).
2. Regulation of Iron Homeostasis to Reduce Catalytic Iron Levels: Iron is the catalytic core of ferroptosis, and estrogen reduces the intracellular labile iron pool through various means. In macrophages within AS plaques, E2 can upregulate the expression of the iron export protein FPN1 via an ERα-dependent mechanism, accelerating iron efflux and thus effectively preventing iron overload-induced ferroptosis (Ozkan and Bakar-Ates, 2023). Concurrently, estrogen may also reduce the release of stored iron by inhibiting NCOA4-mediated ferritinophagy. This regulation of iron homeostasis is crucial for limiting the formation of the necrotic core within the plaque.
3. Direct Chemical Antioxidant Activity: The A-ring of the E2 molecule itself is a phenolic hydroxyl structure, which gives it the potential to act as a chain-breaking antioxidant capable of directly scavenging lipid peroxyl radicals. This direct chemical neutralization can serve as an important backup defense when major enzymatic antioxidant systems are compromised.

### 3.2 Potential multi-dimensional pro-ferroptotic effects of androgens on vascular cells

In contrast to the protective effects of estrogen, some evidence suggests that androgens may be a potential risk factor for promoting AS progression in men, a mechanism that may involve increasing the susceptibility of vascular cells to ferroptosis.

1. Exacerbation of Oxidative Stress and Lipid Peroxidation: Androgens, via their receptor AR, can upregulate the expression and activity of NADPH oxidase (NOX) family subunits, leading to increased intracellular reactive oxygen species (ROS) production (Costa et al., 2015; Alves et al., 2024). Excessive ROS not only directly attacks cell membrane lipids but also consumes intracellular GSH, thereby indirectly weakening the defensive function of the GPX4 system and making cells more prone to ferroptosis.
2. Potential Promotion of Iron Uptake and Iron Homeostasis Imbalance: Men generally have higher systemic iron stores than women. At the cellular level, it is theoretically speculated that androgens may upregulate TFR1 expression via AR, thereby enhancing the cell’s capacity for iron uptake (Campesi et al., 2012). In the context of a higher systemic iron load in men, this cell-autonomous increase in iron absorption would significantly elevate the risk of iron overload and ferroptosis in cells within AS plaques.

### 3.3 Mitochondria: the core battlefield for sex hormone regulation of ferroptosis

Mitochondria are not only the primary sites of cellular energy metabolism but also major sources of reactive oxygen species (ROS) production and key hubs of iron metabolism, making them a central locus in determining whether a cell undergoes ferroptosis. Sex hormones, particularly estrogen, constitute another critical line of defense in inhibiting vascular cell ferroptosis and delaying AS progression by precisely regulating mitochondrial biogenesis, functional homeostasis, and quality control.

#### 3.3.1 Estrogen maintains mitochondrial health via the PGC-1α signaling axis, inhibiting ferroptosis

One of the core mechanisms of estrogen’s protective effect on mitochondria is through the activation of peroxisome proliferator-activated receptor-gamma coactivator 1-alpha (PGC-1α), a master regulator of mitochondrial biogenesis. In vascular cells, 17β-estradiol (E2) can upregulate the expression and activity of PGC-1α in an estrogen receptor α (ERα)-dependent manner (Yang et al., 2019; Kemper et al., 2014). Activated PGC-1α, in turn, initiates its downstream targets, nuclear respiratory factor 1 (NRF-1) and mitochondrial transcription factor A (TFAM), to synergistically promote mitochondrial DNA replication and transcription. This increases the number and function of healthy mitochondria, thereby enhancing the cell’s oxidative phosphorylation capacity and ATP production efficiency (Mattingly et al., 2008). This process not only improves the cell’s ability to meet energy demands but, more importantly, a well-functioning mitochondrial respiratory chain effectively reduces electron leakage and ROS production, thus mitigating the risk of lipid peroxidation at its source. Furthermore, estrogen can also induce a form of alternative, Rab9-mediated mitophagy that is independent of the classic LC3 pathway, via the SIRT1/LKB1/AMPK pathway. This allows for the timely clearance of dysfunctional or damaged mitochondria, preventing them from releasing pro-apoptotic factors or becoming catalytic centers for ferroptosis (Sasaki et al., 2021). This dual regulation of both the “quality” and “quantity” of mitochondria collectively builds a robust anti-ferroptotic defense system and is a key reason for the resilience that estrogen confers upon vascular cells.

#### 3.3.2 Androgens and mitochondrial dysfunction: a potential driver of ferroptosis

In contrast to the protective effects of estrogen, abnormal levels of androgens (either too high or too low) are closely associated with mitochondrial dysfunction and oxidative stress. Clinical studies have found that low testosterone levels in male patients with type 2 diabetes are significantly correlated with increased mitochondrial ROS production, decreased mitochondrial membrane potential, and elevated levels of oxidative stress markers, all of which are favorable conditions for ferroptosis (Rovira-Llopis et al., 2017). Animal experiments have further shown that exogenous supplementation with synthetic androgens inhibits the vasoprotective mitochondrial adaptive changes induced by exercise, including the suppression of PGC-1α expression, while exacerbating oxidative stress (Sun et al., 2013). Of particular concern is that perinatal exposure to testosterone can have long-term effects on endothelial progenitor cells through epigenetic mechanisms such as DNA methylation. This leads to sustained suppression of the expression of ERβ and key mitochondrial antioxidant enzymes (e.g., SOD2) in the endothelial cells they differentiate into, thereby increasing the risk of vascular dysfunction in adulthood (Xie et al., 2017). Therefore, androgens may, through direct or indirect means, impair mitochondrial function and weaken their ability to combat oxidative stress, thus making vascular cells more susceptible to damage like ferroptosis. This may be a significant cell-intrinsic mechanism contributing to the earlier onset and higher incidence of AS in men.

## 4 Sex differences in traditional cardiovascular risk factors and their potential association with ferroptosis

Beyond the direct regulation by sex hormones, sex differences in AS are also manifested in the distribution and effects of traditional risk factors. These factors may have as-yet-unclarified interactions with the ferroptosis pathway.

### 4.1 Sex dimorphism in lipid metabolism and its impact on ferroptosis substrates

For a long time, low-density lipoprotein cholesterol (LDL-C) has been considered a core risk factor for AS. However, growing evidence suggests that risk thresholds based on men may not accurately assess cardiovascular risk in women, particularly premenopausal women. Before puberty, female children already have slightly but significantly higher levels of total cholesterol (TC) and LDL-C than male children, suggesting an intrinsic metabolic difference independent of adult sex hormone levels (Kafol et al., 2025). After sexual maturation, estrogen exerts a cardioprotective effect by upregulating the LDL receptor, promoting reverse cholesterol transport, and thus effectively lowering LDL-C and raising high-density lipoprotein cholesterol (HDL-C). However, this favorable lipid profile rapidly reverses after menopause. More importantly, some emerging lipid markers, such as remnant cholesterol (RC) and the atherogenic index of plasma (AIP), show greater sex-specificity than traditional markers. For example, AIP levels are significantly and positively correlated with testosterone deficiency in adult men (Tai et al., 2025), while the neutrophil-to-HDL-C ratio (NHR), an indicator that combines inflammation and lipid metabolism, is an independent predictor of coronary artery disease severity only in male ACS patients (Wang C. et al., 2025). These sex differences in lipid metabolism may ultimately modulate the susceptibility of vascular cells to ferroptosis by directly altering the composition and abundance of polyunsaturated fatty acids (PUFAs) in cell membranes, thereby changing the substrate pool for lipid peroxidation.

### 4.2 Adipose tissue distribution and menopausal transition

The distribution pattern of adipose tissue is another key sex-differentiated factor. Premenopausal women tend to store fat subcutaneously (a “pear-shaped” body), a type of fat with better metabolic properties. In contrast, men are more prone to storing fat in the abdominal viscera (an “apple-shaped” body). Visceral adipose tissue (VAT) is a highly active endocrine organ that secretes large amounts of pro-inflammatory cytokines (e.g., TNF-α, IL-6) and adipokines, directly promoting insulin resistance and vascular inflammation. After menopause, as estrogen levels decline, women experience a significant centripetal redistribution of fat, with an increase in visceral fat. This shift is a major reason for the sharp increase in their cardiovascular risk (Sánchez-García et al., 2025). This chronic, low-grade inflammatory state driven by adipose tissue not only provides a permissive environment for the development of AS but may also indirectly lower the threshold for vascular cells (such as endothelial cells and macrophages) to resist ferroptosis through systemic oxidative stress.

### 4.3 Progesterone and gonadotropins: overlooked regulators

In the context of the “sex hormone-ferroptosis” regulatory axis, beyond the core roles of estrogen and androgen, progesterone and its upstream regulators—luteinizing hormone (LH) and follicle-stimulating hormone (FSH)—also play pivotal roles. Through direct or indirect mechanisms, these hormones significantly contribute to the sexual dimorphism of AS and potentially interact with the ferroptosis pathway.

#### 4.3.1 Progesterone: duality of its mechanism of action

The biological effects of progesterone in the cardiovascular system exhibit a notable duality, with existing experimental evidence revealing potentially contradictory regulatory patterns. On one hand, research indicates that progesterone may upregulate the expression of hepcidin, thereby indirectly interfering with the stability of the iron export protein FPN1 and promoting intracellular iron accumulation, which lays the groundwork for ferroptosis activation (Li et al., 2016). This mechanism might explain why combined estrogen-progestin replacement therapy has failed to achieve the expected cardiovascular protective effects in some clinical trials. On the other hand, epidemiological studies suggest that progesterone may also exert protective effects. A cohort study in elderly men found that serum progesterone concentrations were negatively correlated with the extent of carotid atherosclerosis (Ma et al., 2009). These conflicting findings suggest that the effects of progesterone are highly dependent on host factors such as sex, age, hormone receptor expression levels, and synergistic or antagonistic interactions with other sex hormones.

#### 4.3.2 Gonadotropins (LH and FSH): extending from reproductive regulation to vascular effects

LH and FSH, traditionally considered core regulators of the reproductive axis, have recently been shown to have direct effects on vascular biology.

Elevated levels of LH have been established as an independent risk factor for AS. Experiments in animal models have confirmed that high LH levels accelerate the formation of atherosclerotic plaques through a molecular mechanism involving the inhibition of the PI3K/Akt/eNOS signaling pathway. This reduces nitric oxide (NO) production in endothelial cells and leads to endothelial dysfunction—a key initiating event in both ferroptosis and AS (Meng et al., 2019).

The role of FSH, however, appears to be highly context-dependent. Vascular endothelial cells and macrophages both express FSH receptors (FSHR), allowing them to respond directly to FSH signals (Li et al., 2017). *In vitro* and *in vivo* studies have shown that FSH can activate the NF-κB signaling pathway, upregulate the expression of adhesion molecules such as vascular cell adhesion molecule-1 (VCAM-1) (Piao et al., 2022), and induce macrophages to release pro-inflammatory cytokines like IL-1β (Han et al., 2023), thereby amplifying the vascular inflammatory response and promoting AS progression. However, the correlation between FSH and cardiovascular events remains inconsistent in clinical cohorts, with some observational studies even reporting an association between lower FSH levels and increased cardiovascular risk in postmenopausal women (Wang et al., 2017). This suggests that the effects of FSH may be profoundly modulated by external factors such as estrogen levels.

**FIGURE 2:**
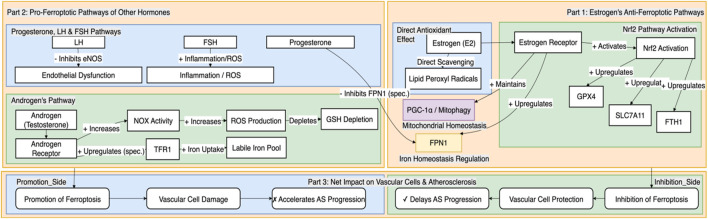
Differential regulation of vascular cell ferroptosis by sex hormones and gonadotropins. This figure systematically illustrates how different sex-related hormones differentially regulate molecular pathways associated with ferroptosis, thereby producing a net inhibitory or promotional effect on the progression of AS. On one hand, estrogen exerts a vasoprotective effect by synergistically inhibiting ferroptosis through multiple pathways, including the activation of the central antioxidant system driven by Nrf2, the optimization of cellular iron homeostasis by upregulating FPN1, and the maintenance of mitochondrial function and homeostasis. These mechanisms collectively delay the progression of AS. On the other hand, androgens may promote ferroptosis by increasing oxidative stress and potentially upregulating TFR1 to enhance cellular iron uptake. Furthermore, progesterone, LH, and FSH are also involved in promoting ferroptosis through mechanisms such as potentially interfering with FPN1 stability, inhibiting eNOS to cause endothelial dysfunction, and exacerbating inflammation and oxidative stress, respectively, which together accelerate the pathological progression of AS. This regulatory network provides a key molecular framework for understanding the sex differences in AS.

In summary, progesterone and gonadotropins regulate oxidative stress, endothelial integrity, and inflammatory cascades, forming a complex network that is intertwined with the ferroptosis pathway. Integrating these hormones into the analytical framework of the “sex hormone-ferroptosis regulatory axis” is crucial for a comprehensive understanding of the sex differences in AS, as illustrated in Figure 2.

## 5 Discussion and outlook

This section aims to systematically evaluate the value of the “sex hormone-ferroptosis regulatory axis” theoretical framework, its current challenges, and future directions, thereby demonstrating its significant implications for understanding sex differences in AS and developing precision medicine.

### 5.1 New theoretical framework unifies clinical observations and molecular mechanisms, and offers a “window of opportunity” reinterpretation for the HRT controversy

The “sex hormone-ferroptosis regulatory axis” theoretical framework, as constructed and expanded in this review, more tightly connects macroscopic clinical epidemiological observations (sex differences in AS, menopausal risk surge) with contemporary cell death molecular mechanisms (ferroptosis) by incorporating the regulatory roles of progesterone and gonadotropins. This provides a concrete and testable molecular entity to explain the long-standing clinical problem of sex differences in AS. More importantly, this framework provides a novel molecular perspective for reinterpreting the controversial results of hormone replacement therapy (HRT) clinical trials, namely, the “window of opportunity” hypothesis: the heterogeneous efficacy of HRT may critically depend on the timing of intervention. Estrogen supplementation in the early perimenopausal period may exert protective effects by inhibiting ferroptosis, whereas its effect in late-stage AS may be minimal. This highlights another core significance of this review: to drive a paradigm shift in AS research from a focus on macroscopic risk factors to more microscopic, sex-specific molecular targeting strategies. For example, the nuclear receptors CAR and PXR, as cardiovascular metabolic regulators, have garnered attention for their roles in lipid and glucose homeostasis, blood pressure regulation, and atherosclerosis (Hukkanen et al., 2025). Research has found that CAR activation can lower serum triglycerides and cholesterol and possesses anti-atherosclerotic properties, suggesting the potential therapeutic value of targeting hormone receptors in cardiovascular diseases.

### 5.2 Current research challenges and limitations

Although the “sex hormone-ferroptosis regulatory axis” offers a promising theoretical framework for understanding sex differences in AS, we must acknowledge the significan challenges and inherent limitations of current research. First, the molecular mechanistic evidence chain is incomplete; many key links (such as the direct regulation of iron uptake molecules by androgens) still rely on indirect evidence or inference and urgently require experimental validation. Second, a significant translational gap exists in current animal models, largely due to a long-standing “male bias” in cardiovascular research. The vast majority of preclinical studies have used only male animals, which has led to a poor understanding of the unique pathophysiology of AS in female animals, severely limiting the generalizability and clinical translation of research findings to the female population. For example, studies on Qsox1’s response to hypertension in vascular remodeling (Sadoune et al., 2025) and the role of vascular smooth muscle cells in vascular remodeling and plaque stability (Alam et al., 2025), while revealing the complexity of vascular biology, also highlight the challenges of extrapolating animal model results to humans. A study of 500 school-aged children aged 10–17 showed significant differences in physical fitness indicators such as grip strength, standing long jump, and maximal oxygen uptake between males and females (Manzoor et al., 2025), underscoring the importance of considering sex differences in animal models. Finally, complex multifactorial interactions pose a major challenge. Genetic background, lifestyle (especially dietary iron), and the gut microbiome all interact with the sex hormone and ferroptosis networks, making it extremely difficult to precisely dissect the independent contribution of sex hormones. For example, changes in the gut microbiota in the development of metabolic syndrome in the elderly (Ivanova et al., 2025) reveal how multiple factors collectively influence disease progression.

### 5.3 Future research needs to utilize multi-omics and new models to elucidate mechanisms and develop biomarkers for clinical translation of sex-specific therapies

To address these challenges and realize the vision of precision medicine proposed in this review, future research should focus on multi-dimensional exploration. At the basic research level, single-cell multi-omics and spatial transcriptomics technologies should be used to map the sex-differentiated molecular blueprint of human AS plaques, and cell-type-specific gene-edited animal models should be developed to precisely parse the autonomous role of hormones. This will directly address the goals of “deciphering the regulatory axis” and “identifying druggable targets” outlined in the introduction. For example, c-Met as a potential target for cardiovascular diseases (Gao et al., 2025) and the application of radiolabeled fibroblast activation protein inhibitors (FAPI) in non-tumor molecular imaging (Jafari et al., 2025) both offer new directions for future precision medicine. At the clinical translation level, there is an urgent need to develop and validate non-invasive circulating biomarkers that can reflect the *in vivo* state of ferroptosis, and ultimately to promote clinical trials for novel hormone receptor modulators, highly effective ferroptosis inhibitors, and other sex-specific therapeutic regimens. For example, miRNA biosensors for cardiovascular diseases (Vafadar et al., 2025) offer the possibility of non-invasively detecting ferroptosis-related biomarkers. Additionally, studies exploring the sex-specific association between serum phospholipid very-long-chain saturated fatty acids and cognitive function in the elderly have found that higher levels of VLSFAs are associated with better cognitive function in women (Lin et al., 2025), highlighting the importance of sex-specificity in biomarker research. This is the essential path to translating the theoretical framework into clinical practice and achieving precision medicine.

## 6 Conclusion

This review has systematically constructed and thoroughly elaborated on the pivotal role of the “sex hormone-ferroptosis regulatory axis” in modulating the sex differences in atherosclerosis. The core argument is that estrogen acts as an endogenous, multi-target inhibitor of ferroptosis, conferring robust anti-ferroptotic capabilities to vascular cells by activating the Nrf2 central antioxidant system, optimizing cellular iron homeostasis (e.g., upregulating FPN1 and inhibiting ferritinophagy), and exerting direct antioxidant effects. This collectively builds a strong cardiovascular protective barrier in premenopausal women. Conversely, androgens appear to act as endogenous promoters of ferroptosis, potentially increasing the susceptibility of vascular cells to ferroptosis through multiple mechanisms, including amplifying oxidative stress, exacerbating cellular iron accumulation, and promoting the generation of lipid substrates, thereby accelerating the pathological progression of AS in men.

The establishment of this theoretical framework not only provides a novel, cell-death-centered molecular perspective for a deeper understanding of the long-standing sex differences in atherosclerosis but also offers new insights for re-examining and interpreting the complex and often contradictory results of past hormone replacement therapy clinical trials. More importantly, it uncovers a series of potential, sex-specific new therapeutic targets, laying a solid theoretical foundation for the future transition from a traditional “one-size-fits-all” model to an era of individualized, sex-stratified precision cardiovascular medicine.

## References

[B1] Alam P. Tharp D. L. Bowles H. J. Grisanti L. A. Bui H. Bender S. B. (2025). Genetic silencing of K(Ca)3.1 inhibits atherosclerosis in ApoE null mice. Channels (Austin). 19 (1), 2538864. 10.1080/19336950.2025.2538864 40753563 PMC12320860

[B2] Alves J. V. da Costa R. M. Awata W. Bruder-Nascimento A. Singh S. Tostes R. C. (2024). NADPH oxidase 4-derived hydrogen peroxide counterbalances testosterone-induced endothelial dysfunction and migration. Am. J. Physiol. Endocrinol. Metab. 327 (1), E1–E12. 10.1152/ajpendo.00365.2023 38690939 PMC11390122

[B3] Cai Z. Deng L. Fan Y. Ren Y. Ling Y. Tu J. (2023). Dysregulation of ceramide metabolism is linked to iron deposition and activation of related pathways in the aorta of atherosclerotic miniature pigs. Antioxidants (Basel) 13 (1), 4. 10.3390/antiox13010004 38275624 PMC10812416

[B4] Campesi I. Sanna M. Zinellu A. Carru C. Rubattu L. Bulzomi P. (2012). Oral contraceptives modify DNA methylation and monocyte-derived macrophage function. Biol. Sex. Differ. 3, 4. 10.1186/2042-6410-3-4 22284681 PMC3298494

[B5] Chen X. Niu B. Zhang Z. Zhang B. Xue T. Liu Y. (2025). Low-molecular-weight fucoidan ameliorates ferroptosis in diabetic kidney disease through SLC7A11/GSH/GPX4 activation. Fitoterapia 185, 106779. 10.1016/j.fitote.2025.106779 40752796

[B6] Costa T. J. Ceravolo G. S. dos Santos R. A. de Oliveira M. A. Araújo P. X. Giaquinto L. R. (2015). Association of testosterone with estrogen abolishes the beneficial effects of estrogen treatment by increasing ROS generation in aorta endothelial cells. Am. J. Physiol. Heart Circ. Physiol. 308 (7), H723–H732. 10.1152/ajpheart.00681.2014 25637546

[B7] Cui L. Lu M. Li W. Yin Y. Li C. Chen M. (2025). Hu Gan Tang ameliorates metabolic-associated fatty liver disease by inhibiting ferroptosis through the Nrf2/GPX4 pathway. J. Ethnopharmacol. 353 (Pt A), 120342. 10.1016/j.jep.2025.120342 40752599

[B8] Deng Y. Lin X. Wei J. Chen B. Yan H. Wang B. (2025). Endothelial cell iron overload and ferroptosis mediate thrombosis and inflammation through the miR-32-5p/neurofibromin 2 pathway. Eur. J. Med. Res. 30 (1), 463. 10.1186/s40001-025-02716-y 40481609 PMC12142838

[B9] Dixon S. J. Lemberg K. M. Lamprecht M. R. Skouta R. Zaitsev E. M. Gleason C. E. (2012). Ferroptosis: an iron-dependent form of nonapoptotic cell death. Cell 149 (5), 1060–1072. 10.1016/j.cell.2012.03.042 22632970 PMC3367386

[B10] Doll S. Proneth B. Tyurina Y. Y. Panzilius E. Kobayashi S. Ingold I. (2017). ACSL4 dictates ferroptosis sensitivity by shaping cellular lipid composition. Nat. Chem. Biol. 13 (1), 91–98. 10.1038/nchembio.2239 27842070 PMC5610546

[B11] Donovan A. Brownlie A. Zhou Y. Shepard J. Pratt S. J. Moynihan J. (2000). Positional cloning of zebrafish ferroportin1 identifies a conserved vertebrate iron exporter. Nature 403 (6771), 776–781. 10.1038/35001596 10693807

[B12] Gao J. Wang X. Zhang L. Wu C. Xu H. Xie M. (2025). Unveiling the role of c-Met: a promising target for cardiovascular disease. Pharmacol. Res. 219, 107893. 10.1016/j.phrs.2025.107893 40754043

[B13] Han J. L. Song Y. X. Yao W. J. Zhou J. Du Y. Xu T. (2023). Follicle-stimulating hormone provokes macrophages to secrete IL-1β contributing to atherosclerosis progression. J. Immunol. 210 (1), 25–32. 10.4049/jimmunol.2200475 36368721

[B14] Hu Z. Zhao M. Shen H. Wei L. Sun J. Gao X. (2026). Organelle symphony: nuclear factor erythroid 2-related factor 2 and nuclear factor-kappa B in stroke pathobiology. Neural Regen. Res. 21 (4), 1483–1496. 10.4103/NRR.NRR-D-24-01404 40146002 PMC12407511

[B15] Hukkanen J. Küblbeck J. Hakkola J. Rysä J. (2025). Nuclear receptors CAR and PXR as cardiometabolic regulators. Pharmacol. Res. 219, 107892. 10.1016/j.phrs.2025.107892 40752780

[B16] Ivanova E. M. Kosmina K. O. Shumkov A. U. Zabezhinsky A. M. Mayorova A. M. Raevskii K. P. (2025). The role of changes in the intestinal microbiota in the development of metabolic syndrome in the elderly (literature review). Adv. Gerontol. 38 (2), 243–250. 40753560

[B17] Jafari E. Juweid M. E. Bahtouee M. Pourbehi M. Esmaeilinejad K. Jokar N. (2025). Beyond cancer: the role of radiolabeled fibroblast activation protein inhibitors (FAPI) in non-oncological molecular imaging. Acad. Radiol. (25). 10.1016/j.acra.2025.07.034 40753024

[B18] Jinson S. Zhang Z. Lancaster G. I. Murphy A. J. Morgan P. K. (2025). Iron, lipid peroxidation, and ferroptosis play pathogenic roles in atherosclerosis. Cardiovasc Res. 121 (1), 44–61. 10.1093/cvr/cvae270 39739567

[B19] Kafol J. Becker M. Cugalj K. B. Sikonja J. Mlinaric M. Sedej K. (2025). Sex differences in cholesterol levels among prepubertal children. Atherosclerosis, 120484. 10.1016/j.atherosclerosis.2025.120484 40841242

[B20] Karbakhsh Ravari F. Ghasemi Gorji M. Rafiei A. (2025). From iron-driven cell death to clot formation: the emerging role of ferroptosis in thrombogenesis. Biomed. Pharmacother. 189, 118328. 10.1016/j.biopha.2025.118328 40628161

[B21] Kemper M. F. Stirone C. Krause D. N. Duckles S. P. Procaccio V. (2014). Genomic and non-genomic regulation of PGC1 isoforms by estrogen to increase cerebral vascular mitochondrial biogenesis and reactive oxygen species protection. Eur. J. Pharmacol. 723, 322–329. 10.1016/j.ejphar.2013.11.009 24275351 PMC4028038

[B22] Li X. Rhee D. K. Malhotra R. Mayeur C. Hurst L. A. Ager E. (2016). Progesterone receptor membrane component-1 regulates hepcidin biosynthesis. J. Clin. Invest 126 (1), 389–401. 10.1172/JCI83831 26657863 PMC4701562

[B23] Li X. Chen W. Li P. Wei J. Cheng Y. Liu P. (2017). Follicular stimulating hormone accelerates atherogenesis by increasing endothelial VCAM-1 expression. Theranostics 7 (19), 4671–4688. 10.7150/thno.21216 29187895 PMC5706091

[B24] Li D. Mao Y. Zhang X. Wang Y. Tang H. Huang H. (2025). Epigallocatechin-3-Gallate promotes recanalization in deep vein thrombosis by modulating endothelial progenitor cell ferroptosis through the Nrf2 pathway. Phytother. Res. 39 (3), 1632–1644. 10.1002/ptr.8457 39918021

[B25] Lin G. Tang J. Pang K. Liu X. Zhou L. Li B. (2025). Sex-specific association of serum phospholipid very-long-chain saturated fatty acids with cognitive performance among elderly adults. Clin. Nutr. 52, 103–112. 10.1016/j.clnu.2025.07.016 40752036

[B26] Ma Q. Sun X. Chen Y. Chen X. Zhi G. Tan G. (2009). Progesterone levels and carotid intima-media thickness: a negative association in older northern Chinese men. Tex Heart Inst. J. 36 (4), 303–308. 19693303 PMC2720297

[B27] Mancias J. D. Wang X. Gygi S. P. Harper J. W. Kimmelman A. C. (2014). Quantitative proteomics identifies NCOA4 as the cargo receptor mediating ferritinophagy. Nature 509 (7498), 105–109. 10.1038/nature13148 24695223 PMC4180099

[B28] Manzoor S. Hassan D. Khalid S. Irshad M. H. Salik S. (2025). Age and gender specific normative physical fitness values among school going children: a cross-sectional analytical study. J. Pak Med. Assoc. 75 (7), 1067–1071. 10.47391/JPMA.20600 40751614

[B29] Mattingly K. A. Ivanova M. M. Riggs K. A. Wickramasinghe N. S. Barch M. J. Klinge C. M. (2008). Estradiol stimulates transcription of nuclear respiratory factor-1 and increases mitochondrial biogenesis. Mol. Endocrinol. 22 (3), 609–622. 10.1210/me.2007-0029 18048642 PMC2262171

[B30] Meng X. Li X. Xu X. Li P. Chen Y. Fu X. (2019). Elevated luteinizing hormone contributes to atherosclerosis formation by inhibiting nitric oxide synthesis via PI3K/Akt pathway. Vasc. Pharmacol. 121, 106582. 10.1016/j.vph.2019.106582 31437529

[B31] Min X. Du Z. Wei J. Yuan Z. She Y. Jin X. (2025). Pyridoxal phosphate inhibits alpha-synuclein-induced ferroptosis by activating GOT1 to enhance the methionine salvage pathway in Parkinson's disease. Exp. Neurol. 393, 115411. 10.1016/j.expneurol.2025.115411 40752725

[B32] Ozkan E. Bakar-Ates F. (2023). Etoposide in combination with erastin synergistically altered iron homeostasis and induced ferroptotic cell death through regulating IREB2/FPN1 expression in estrogen receptor positive-breast cancer cells. Life Sci. 312, 121222. 10.1016/j.lfs.2022.121222 36442526

[B33] Peng J. Zou M. Zhang Q. Liu D. Chen S. Fang R. (2025). Symphony of regulated cell death: unveiling therapeutic horizons in sarcopenia. Metabolism 172, 156359. 10.1016/j.metabol.2025.156359 40752569

[B34] Peng X. Sun B. Tang C. Shi C. Xie X. Wang X. (2025). HMOX1-LDHB interaction promotes ferroptosis by inducing mitochondrial dysfunction in foamy macrophages during advanced atherosclerosis. Dev. Cell 60 (7), 1070–1086.e8. 10.1016/j.devcel.2024.12.011 39731912

[B35] Piao J. Yin Y. Zhao Y. Han Y. Zhan H. Luo D. (2022). Follicle-stimulating hormone accelerates atherosclerosis by activating PI3K/akt/NF-κB pathway in mice with androgen deprivation. J. Vasc. Res. 59 (6), 358–368. 10.1159/000527239 36412620

[B36] Probst L. Dächert J. Schenk B. Fulda S. (2017). Lipoxygenase inhibitors protect acute lymphoblastic leukemia cells from ferroptotic cell death. Biochem. Pharmacol. 140, 41–52. 10.1016/j.bcp.2017.06.112 28595877

[B37] Qiu Q. Sun Q. Yang J. Yuan Q. Wang P. Liu Q. (2025). The molecular mechanism by which CTSB degrades FPN to disrupt macrophage iron homeostasis and promote the progression of atherosclerosis. Mol. Cell Biochem. 480 (6), 3889–3906. 10.1007/s11010-025-05228-9 39960586

[B38] Rovira-Llopis S. Bañuls C. de Marañon A. M. Diaz-Morales N. Jover A. Garzon S. (2017). Low testosterone levels are related to oxidative stress, mitochondrial dysfunction and altered subclinical atherosclerotic markers in type 2 diabetic male patients. Free Radic. Biol. Med. 108, 155–162. 10.1016/j.freeradbiomed.2017.03.029 28359952

[B39] Sadoune M. Mourad J. Luc C. Ragot H. Mateo P. Polidano E. (2025). Qsox1 contributes to vascular remodelling in response to hypertension. J. Vasc. Res., 1–15. 10.1159/000546331 40753967

[B40] Sakamuri A. Visniauskas B. Kilanowski-Doroh I. McNally A. B. Imulinde A. Kamau A. (2024). Testosterone deficiency promotes arterial stiffening independent of sex chromosome complement. Biol. Sex. Differ. 15 (1), 46. 10.1186/s13293-024-00624-0 38845040 PMC11155160

[B41] Sánchez-García M. León-Wu K. de Miguel-Ibáñez R. López-Juárez N. Ramírez-Rentería C. Espinosa-Cárdenas E. (2025). Metabolic changes in patients with premature ovarian insufficiency: adipose tissue focus-A narrative review. Metabolites 15 (4), 242. 10.3390/metabo15040242 40278371 PMC12029191

[B42] Sasaki Y. Ikeda Y. Uchikado Y. Akasaki Y. Sadoshima J. Ohishi M. (2021). Estrogen plays a crucial role in rab9-dependent mitochondrial autophagy, delaying arterial senescence. J. Am. Heart Assoc. 10 (7), e019310. 10.1161/JAHA.120.019310 33719502 PMC8174372

[B43] Souza F. R. Rochitte C. E. Silva D. C. Sampaio B. Passarelli M. Santos M. R. D. (2023). Coronary inflammation by computed tomography pericoronary fat attenuation and increased cytokines in young male anabolic androgenic steroid users. Arq. Bras. Cardiol. 120 (11), e20220822. 10.36660/abc.20220822 37991119 PMC10697680

[B44] Sun M. Shen W. Zhong M. Wu P. Chen H. Lu A. (2013). Nandrolone attenuates aortic adaptation to exercise in rats. Cardiovasc Res. 97 (4), 686–695. 10.1093/cvr/cvs423 23338851

[B45] Swahn E. Sederholm Lawesson S. Alfredsson J. Fredrikson M. Angerås O. Duvernoy O. (2024). Sex differences in prevalence and characteristics of imaging-detected atherosclerosis: a population-based study. Eur. Heart J. Cardiovasc Imaging 25 (12), 1663–1672. 10.1093/ehjci/jeae217 39158095 PMC11601724

[B46] Tai Y. Chen B. Kong Y. Shang J. (2025). Association between the atherogenic index of plasma and testosterone deficiency in American adults: a cross-sectional study from NHANES 2011-2016. Front. Endocrinol. (Lausanne) 16, 1531221. 10.3389/fendo.2025.1531221 40405972 PMC12094991

[B47] Tan Q. M. Li M. Zhu J. M. Liao B. Z. Kong L. Y. Luo J. G. (2025). Surface plasmon resonance guided identification of quinolone alkaloids from the fruits of tetradium ruticarpum as FSP1 inhibitors. J. Nat. Prod. 88 (8), 1919–1927. 10.1021/acs.jnatprod.5c00595 40748910

[B48] Vafadar A. Alavimanesh S. Babadi S. Hosseinpour V. Ehtiati S. Savardashtaki A. (2025). MicroRNA biosensors for cardiovascular disease detection. Clin. Chim. Acta. 578, 120525. 10.1016/j.cca.2025.120525 40752880

[B49] Wang N. Shao H. Chen Y. Xia F. Chi C. Li Q. (2017). Follicle-stimulating hormone, its association with cardiometabolic risk factors, and 10-year risk of cardiovascular disease in postmenopausal women. J. Am. Heart Assoc. 6 (9), e005918. 10.1161/JAHA.117.005918 28855169 PMC5634260

[B50] Wang Y. Zhu Q. Chen Z. Wang X. Wang Y. (2025). Self-adaptive covalent coating for vascular stents: coordinated coagulation-inflammation regulation to support re-endothelialization for atherosclerosis control. Biomaterials 325, 123600. 10.1016/j.biomaterials.2025.123600 40753783

[B51] Wang C. Shang K. Cao L. Kuang J. Ning X. Chen H. (2025). Sex differences of neutrophil to high-density lipoprotein cholesterol ratio in predicting the severity of coronary lesions in acute coronary syndrome patients. Lipids Health Dis. 24 (1), 54. 10.1186/s12944-025-02478-w 39962499 PMC11831777

[B52] Ward D. M. Cloonan S. M. (2019). Mitochondrial iron in human health and disease. Annu. Rev. Physiol. 81, 453–482. 10.1146/annurev-physiol-020518-114742 30485761 PMC6641538

[B53] Wu Y. Q. Wu R. M. Zeng Y. Le Xu X. (2025). Nrf2-mediated inhibition of ferroptosis contributes to the amelioration of atherosclerosis by polydatin. Toxicol. Appl. Pharmacol. 504, 117538. 10.1016/j.taap.2025.117538 40886948

[B54] Xie W. Ren M. Li L. Zhu Y. Chu Z. Zhu Z. (2017). Perinatal testosterone exposure potentiates vascular dysfunction by ERβ suppression in endothelial progenitor cells. PLoS One 12 (8), e0182945. 10.1371/journal.pone.0182945 28809938 PMC5557363

[B55] Xu X. Xu X. D. Ma M. Q. Liang Y. Cai Y. B. Zhu Z. X. (2024). The mechanisms of ferroptosis and its role in atherosclerosis. Biomed. Pharmacother. 171, 116112. 10.1016/j.biopha.2023.116112 38171246

[B56] Yang Q. Wang C. Jin Y. Ma X. Xie T. Wang J. (2019). Disocin prevents postmenopausal atherosclerosis in ovariectomized LDLR-/- mice through a PGC-1α/ERα pathway leading to promotion of autophagy and inhibition of oxidative stress, inflammation and apoptosis. Pharmacol. Res. 148, 104414. 10.1016/j.phrs.2019.104414 31449974

[B57] Zhang Y. Liu Y. Huang Q. Wang Z. Li Y. Zhang Q. (2025). Decoding the diet-inflammation nexus: ferroptosis as a therapeutic target. Crit. Rev. Food Sci. Nutr., 1–21. 10.1080/10408398.2025.2540044 40782345

[B58] Zhu X. Chen Y. Xu B. Mou J. Wang M. Gu Q. (2025). Targeting KEAP1/NRF2 interaction with oleuropein ameliorates atherosclerosis by inhibiting macrophage ferroptosis. Free Radic. Biol. Med. 240 (25), 566–582. 10.1016/j.freeradbiomed.2025.08.036 40885455

